# Molecular characterization of mitochondrial Amerindian haplogroups and the amelogenin gene in human ancient DNA from three archaeological sites in Lambayeque - Peru

**DOI:** 10.1590/1678-4685-GMB-2019-0265

**Published:** 2020-11-25

**Authors:** Jorge Victor Wilfredo Cachay Wester, Vanny Judith Soplapuco Vilchez, Carlos Eduardo Wester La Torre, Luis Alberto Rodriguez-Delfin

**Affiliations:** 1 Universidade de São Paulo, Faculdade de Medicina de Ribeirão Preto, Departamento de Genética, Laboratório de Genética Humana e Médica, Ribeirão Preto, SP, Brazil.; 2 Universidad Nacional Pedro Ruiz Gallo, Facultad de Ciencias Biológicas, Departamento de Biología, Laboratorio de Genética y Biología Molecular, Lambayeque, Peru.; 3 Museo Nacional de Arqueología y Etnología Brüning, Lambayeque, Peru.; 4 Universidad Nacional Pedro Ruiz Gallo, Facultad de Ciencias Histórico Sociales y Educación, Departamento de Arqueología, Lambayeque, Peru.

**Keywords:** Ancient DNA, *AMEL (X/Y)* locus, Amerindian haplogroup, Peru

## Abstract

Important pre-Inca civilizations, known by their great political and religious structures, inhabited the northern coast of Peru. Archeological and anthropological studies have shown that people from these villages have hierarchical strata, but the genetic structure has been poorly studied. Here, we aimed to perform a molecular characterization of the Amerindian maternal lineages and the amelogenin gene in skeletons collected from three archeological sites in Lambayeque. Ancient DNA (aDNA) samples were analyzed with conventional PCR to assess the nine-base pair (9 bp) deletion corresponding to mitochondrial haplogroup B and the identification of haplogroups A, C, and D were obtained with PCR-RFLP experiments. The sex was characterized via amplification of the *AMEL(X/Y)* locus. Haplogroup frequencies were compared with available data from other ancient and modern civilizations from the Peruvian coast and highlands using statistical methods. Our results showed that haplogroup C had the highest frequency, while haplogroup B showed variable diversity in the analyzed populations. The meta-analysis revealed a positive correlation among some coastal villages. We concluded that ancient populations analyzed in our study showed the presence of four Amerindian mitochondrial haplogroups, which is consistent with previous studies.

## Introduction

Lambayeque is located on the northern coast of Peru. This place was an important socio-political center for ancient pre-Inca civilizations such as Moche (100 C.E.-700 C.E.), Sipan (250 C.E.), Sican (900 C.E.-1100 C.E.), and Lambayeque (Naylamp) (700 C.E.-1375 C.E.). Three ancient empires (Chimu, Wari, and Inca) used these lands to control the exchange of agricultural products through the coast, and the remains of its populations have been studied because of their exceptional mortuary practices (Shimada *et al.* 2004).

Lambayeque people occupied the Jequetepeque valley, Pomac, and Chotuna-Chornancap between 700 and 1375 C.E. (Wester La Torre *et al.* 2014; Klaus *et al.* 2016). This civilization was initially identified as part of the Sican culture, but new archeological and anthropological evidence has pointed to the existence of a well-structured socio-political civilization that preceded Sican, and its rise coincided with the end of Moche culture (Klaus and Tam 2009). The expansion of the Lambayeque civilization toward the Jequetepeque valley also indicated that there was a migration process to the south of the northern coast and to the highlands due to political and economic reasons. Therefore, there is a possibility of admixture with other civilizations (Castillo and Donnan 1994) in such a way that it is important to study the genetic structure of this population. 

The first molecular study in American populations using mitochondrial DNA established the presence of four Amerindian mitochondrial haplogroups named A, B, C, and D (Torroni and Wallace 1995). In South America, these four haplogroups are well distributed. Several studies on mitochondrial haplogroups have been performed among native ancient South-American populations (Merriwether *et al.* 1994; Merriwether *et al.* 1995; Moraga *et al.* 2001; Eshleman *et al.* 2003), which have reported the presence of these four haplogroups.

Molecular analysis of ancient human burials from the central Andes determined that haplogroup B has the highest frequency in Andean populations (Shinoda *et al.* 2006; Fehren-Schmitz *et al.* 2010; Valverde *et al.* 2016). In modern populations, similar haplogroup frequencies are observed in regions located in the central south of Peru and the north of Bolivia (Cabana *et al.* 2014). In addition, haplogroups C and D have been found in samples from the coast and the Amazon (Sandoval *et al.* 2013). 

Mitochondrial DNA analysis of ancient populations from the south coast of Peru revealed diachronic variations in the matrilineal genetic composition of the analyzed area, which correlate with the cultural events that occurred in the period of the development of these civilizations (Fehren-Schmitz *et al.* 2010). Meanwhile, the ancient northern coast populations seem to have a close matrilineal structure (Shimada *et al.* 2004). To investigate the maternal relationships around the ancient Peruvian coast and highlands, we aimed to study the maternal heritage and the amelogenin gene by using ancient DNA obtained from human remains of three archeological sites in Lambayeque.

## Material and Methods

### Samples

**Figure 1 -:**
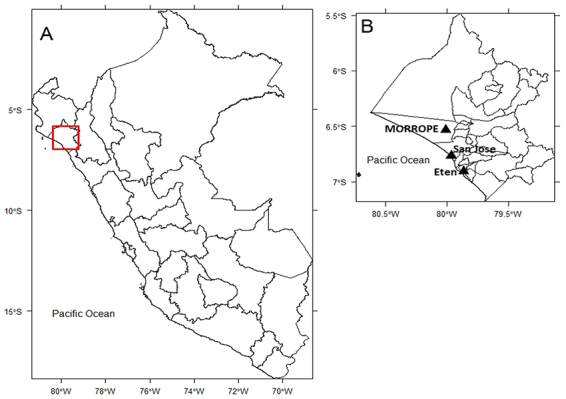
Geographical localization highlighting the three archaeological sites. (A) Political map of Peru; (B) Political map of Lambayeque. Geographical coordinates of latitude and longitude were used to plot the localization of the archaeological sites, represented by black triangles.

**Figure 2 -:**
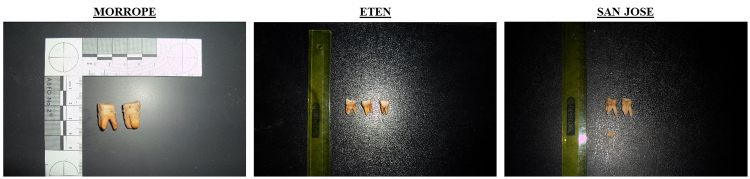
Samples collected in the archaeological sites. Teeth were collected from archaeological sites at Chapel San Pedro de Morrope (MORROPE), Huaca Cascajales (ETEN), and Huaca Tanque Nuevo (SAN JOSE).

Teeth samples from ancient human remains of 32 people buried in three archeological sites located in the Lambayeque valley (Figure 1) were collected at the National Museum of Archaeology and Ethnography Hans Heinrich Brüning, with the permission of the Ministry of Culture from Peru. One of the archeological sites is the Chapel of San Pedro de Morrope, located in the city of Morrope. At this site, there are tombs containing skeletons of several individuals that came from native villages in Lambayeque, belonging to the pre-colonial period. The second site is the Huaca Cascajales located in the city of Eten, which served as a sanctuary for low-middle status ancient people in the late Lambayeque period. The third site is the Huaca Tanque Nuevo located in La Caleta de San Jose town, which was an ancient cemetery used during the Chimu period for local native fishers (Table 1). Skulls and pelvic bone samples from all subjects found at excavations in San Jose and Morrope were used for bioanthropological analysis, and the results were previously published (Klaus *et al.* 2009; Klaus and Tam 2009). Hence, samples from Eten passed through routine analysis at the National Museum of Archaeology and Ethnography Hans Heinrich Brüning. In addition, only teeth samples were used for molecular analysis in this study (Figure 2).

**Table 1 -:** Amerindian haplogroups relative frequencies and Intra-populational Genetic Diversity (*h*_*sk*_ ) in three archeological sites from Lambayeque.

Period	Year (C.E.)	Population	n	Relative frequencies				*h*_*sk*_
				A	B	C	D	
Late Lamb.	1100-1375^a^	Eten	12	0.083	0.25	0.5	0.167	0.7121±0.1053
Chimu	1375-1475^a^	San Jose	5	0.2	0.4	0.4	0	0.8000±0.1640
Pre-colonial	1536-1640^a^	Morrope	15	0	0.333	0.667	0	0.4762±0.0920

a Samples were dating by radiometric techniques. This information was obtained from Klaus *et al.*, 2009; Klaus and Tam, 2009

### Precautions to avoid DNA contamination

Samples were handled by only one person who performed the collection of the samples, pretreatment, and molecular analysis of mitochondrial haplogroups. Full body protective clothing, facemask, and several layers of gloves were worn during the pretreatment. The molecular biology laboratory worked with ancient DNA (aDNA) samples only, and any modern sample was not processed in this laboratory. The DNA extraction, pre-PCR, and post-PCR sample were processed at different work places that were irradiated with UV for at least 40 min and cleaned carefully. Strict workflow protocols for ancient DNA analysis were performed during lab work. Teeth were soaked in a 13% sodium hypochlorite solution for 15 min, rinsed once with ddH2O and 95% ethanol, and dried in a UV-irradiated box. Experiments were performed on duplicate tooth samples from each individual, and the DNA of the person who manipulated the samples was also processed in all the analyses. Negative controls were used during the extraction and PCR experiments. We performed DNA extractions without sample and PCR reactions without DNA.

### DNA extraction

About 1 mm of the dentin layer was removed using a dental drill, and samples were rinsed, soaked, rinsed again, and then dried as described above. Then, the teeth were pulverized in liquid nitrogen and digested in 12 mL of EDTA (0.5 M, pH 8) (PROMEGA, Madison, WI, USA) and 70 µL of proteinase K (100 mg/mL) (PROMEGA) for 18-24 h in a rotating hybridization oven at 55 °C. Samples were centrifuged at 7500 rpm for 10 min and the supernatant was collected in a new autoclaved tube. Genomic DNA was extracted and eluted in a final volume of 100 µL using High Pure DNA Extraction for PCR Kit (Roche, Basel, Switzerland), according to the manufacturer’s instructions. 

### PCR and PCR-RFLP analysis

Mitochondrial DNA regions where the polymorphisms that define the Native American haplogroups and the amelogenin locus are located (*AMEL X/Y*) were amplified by conventional PCR as previously described (Rodriguez-Delfin *et al.* 2001; Morikawa *et al.* 2011). Primer sequences and PCR fragments are shown in the supplementary material (Tables S1-S2 and Figure S1). Polymorphisms were detected by submitting the PCR products to enzymatic reactions for haplogroups A (+663) using *Hae*III (PROMEGA), C (-13259) using *Hinc*II (PROMEGA), and D (-1571) using *Alu*I (PROMEGA). Haplogroup B was detected through the 9 bp deletion. Multiplex PCR was performed to amplify *AMELX* (114 bp) and *AMELY* (120 bp). Amplified and digested fragments were analyzed by non-denaturing 6 % polyacrylamide gel, followed by silver staining (Figure S1). 

### Statistics and meta-analysis

Haplogroup frequencies were obtained using the direct counting method. Hence, relative haplogroup frequencies were computed by mere counting (Tables S3-S5) and the intra-population genetic diversity index (*h*_*sk*_ ) within standard deviations, the fixation index (*F*_*st*_ ), and Nei’s distance (*d*) were determined using Arlequin version 3.5 (Excoffier and Lischer 2010). Haplogroup frequencies were analyzed through Pearson's correlation and principal component analysis (PCA) among populations using R “correlation” (Lüdecke *et al.* 2019) and FactoMineR (Lê *et al.* 2008) packages, respectively. Cluster analysis was performed by comparing the two main clusters in average linkage (UPGMA) using the hclust package in R. In addition, analysis of molecular variance (AMOVA) was performed for each cluster of populations and among all populations using Arlequin version 3.5, in order to obtain the source of variation. Haplogroup frequency data from previously published studies (Fuselli *et al.* 2003; Lewis Jr. *et al.* 2007) were used for statistical analysis.

## Results

Amelogenin gene analysis showed that, of the 32 samples, nine were female and seven were male. The sex could not be characterized for half of the samples. Molecular analysis of the 32 samples resulted in the identification of the four founder Amerindian haplogroups A, B, C, and D. Haplogroup C showed the highest frequency (50%) in Eten. In addition, haplogroup B was present in 25% of this population. Morrope showed frequencies of 66.7% for haplogroup C and 33.3% for haplogroup B. San Jose presented 40% of analyzed samples for each haplogroup B and C. Frequencies for both haplogroups A and D were lower in the three populations. The genetic diversity index was different in Morrope when compared with that in both San Jose and Eten, which suggests potential haplogroup diversity among the Lambayeque villages (Table 1). 

We investigated the mitochondrial haplogroup frequencies of Peruvian ancient and modern civilizations previously reported from the coast and the highlands (Table 2). Correlation analysis showed a strong relationship between two of the northern coast populations analyzed in this study (Eten and Morrope), and we observed that there is a positive correlation with some coastal populations such as Nasca-Rural (Palpa), Nasca-Urban (Palpa), and Middle Horizon (Palpa). This suggests a similar frequency of the four haplogroups among these civilizations. We also observed a negative correlation between our samples and ancient highlands populations studied by Shinoda *et al.* 2006, which divided our data into two sub-groups: one for the coast and another for the highlands (Table 3).

**Table 2 -:** mtDNA haplogroup frequencies in ancient and modern populations from the Peruvian coast and highlands.

Location	Population	Period	n	Haplotype frequencies (%)					Author
				A	B	C	D	others	
Coast	Eten	MH	12	8.3	25	50	16.7	0	This study
	Morrope	LH	15	0	33.3	66.7	0	0	This study
	San Jose	MH	5	20	40	40	0	0	This study
	Ancient north coast	MH	36	19.4	22.2	5.6	30.6	22.2	Shimada *et al.*, 2004
	Paracas (Peninsula)	MH	10	0	0	30	70	0	Fehren-Schmitz *et al.*, 2010
	Paracas (Palpa)	MH	28	7	0	14	79	0	Fehren-Schmitz *et al.*, 2010
	Nasca-Rural (Palpa)	EH	37	2	11	22	65	0	Fehren-Schmitz *et al.*, 2010
	Nasca-Urban (Palpa)	MH	28	0	18	43	39	0	Fehren-Schmitz *et al.*, 2010
	Middle Horizon (Palpa)	MH	11	0	27	36	37	0	Fehren-Schmitz *et al.*, 2010
Highlands	Ancient highlanders	LH	35	8.5	65.7	22.9	2.9	0	Shinoda *et al.*, 2006
	Pacapaccari	MH	16	0	69	31	0	0	Fehren-Schmitz *et al.*, 2010
	Chen Chen	MH	23	39	39	17	4	0	Lewis Jr., Buikstra, & Stone, 2007
	San Martin	M	22	8	55	5	27	5	Fuselli *et al.*, 2003
	Ancash	MH	33	9	52	18	21	0	Lewis Jr. *et al.*, 2007
	Arequipa	M	22	9	68	14	9	0	Fuselli *et al.*, 2003
	Tayacaja	M	60	21	33	30	13	3	Fuselli *et al.*, 2003

*EH: Early Horizon, MH: Middle Horizon, LH: Late Horizon, M: Modern

**Table 3 -:** Pearson correlation among analyzed populations

		1	2	3	4	5	6	7	8	9	10	11	12	13	14	15	16
1	Eten		-	-	-	-	-	-	-	-	-	-	-	-	-	-	-
2	Morrope	0,947^*^		-	-	-	-	-	-	-	-	-	-	-	-	-	-
3	San Jose	0,760	0,838		-	-	-	-	-	-	-	-	-	-	-	-	-
4	Ancient highlanders	-0,707^**^	-0,819^**^	-0,688^**^		-	-	-	-	-	-	-	-	-	-	-	-
5	Ancient north coast	0,283	0,000	-0,324^**^	0,277		-	-	-	-	-	-	-	-	-	-	-
6	Paracas (Peninsula)	0,030	-0,267^**^	-0,485^**^	0,519	0,957^*^		-	-	-	-	-	-	-	-	-	-
7	Paracas (Palpa)	0,234	-0,052^**^	-0,301^**^	0,409	0,978^*^	0,970^*^		-	-	-	-	-	-	-	-	-
8	Nasca-Rural (Palpa)	0,792	0,598	0,267	-0,222^**^	0,796	0,611	0,766		-	-	-	-	-	-	-	-
9	Nasca-Urban (Palpa)	0,774	0,600	0,366	-0,116^**^	0,712	0,554	0,738	0,960^*^		-	-	-	-	-	-	-
10	Middle Horizon (Palpa)	0,437	0,532	0,793	-0,163^**^	-0,334^**^	-0,385^**^	-0,198^**^	0,125	0,362		-	-	-	-	-	-
11	Pacapaccari (Highlanders)	0,545	0,650	0,820	-0,266^**^	-0,285^**^	-0,383^**^	-0,172^**^	0,229	0,444	0,983^*^		-	-	-	-	-
12	Chen Chen	0,165	0,204	0,698	-0,207^**^	-0,518^**^	-0,462^**^	-0,423^**^	-0,255^**^	-0,110^**^	0,654	0,545		-	-	-	-
13	San Martin	0,079	0,057	0,326	0,437	0,008	0,086	0,205	0,135	0,402	0,802	0,737	0,431		-	-	-
14	Ancash	0,414	0,397	0,622	0,101	0,004	-0,010	0,167	0,326	0,570	0,924^*^	0,893	0,538	0,933		-	-
15	Arequipa	0,298	0,368	0,668	0,038	-0,282^**^	-0,282	-0,117^**^	0,079	0,340	0,979^*^	0,938^*^	0,638	0,901^*^	0,957^*^		-
16	Tayacaja	0,757	0,748	**0,953**	-0,508^**^	-0,125^**^	-0,246	-0,069^**^	0,385	0,505	0,804	0,802	0,749	0,472	0,740	0,720	

*Positive correlation

**Negative correlation

**Figure 3 -:**
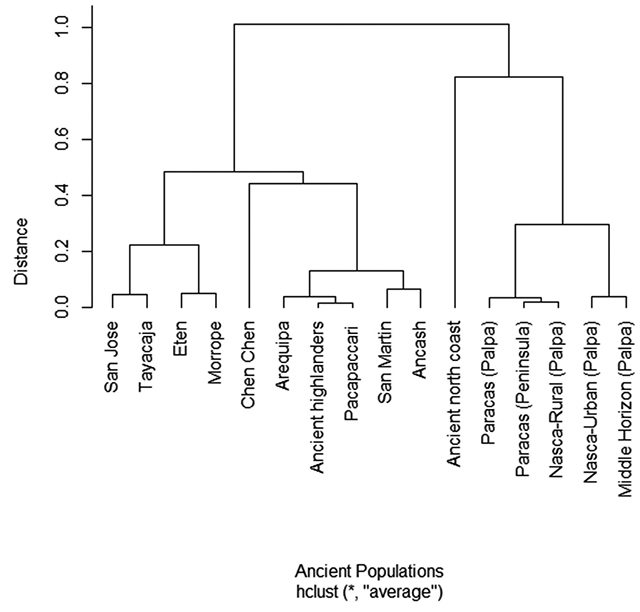
Hierarchical clustering. UPGMA clustering using haplogroup frequencies of 16 ancient populations.

Two main clusters were obtained from hierarchical clustering analysis using a bootstrap with more than 10,000 permutations to ensure the accuracy of the observations (Figure 3). We observed that haplogroup frequencies divided all populations into three sub-groups. The first sub-group comprised our three populations and one population from the highlands (Tayacaja). Indeed, Eten and Morrope have a close genetic distance, but San Jose appears to be closer to a Central Andes population. The second comprised different populations from the south without any cultural relationship. The third grouped the majority of Nazca populations as reported previously, and surprisingly, a northern coast population previously described was incorporated in this cluster.

**Figure 4 -:**
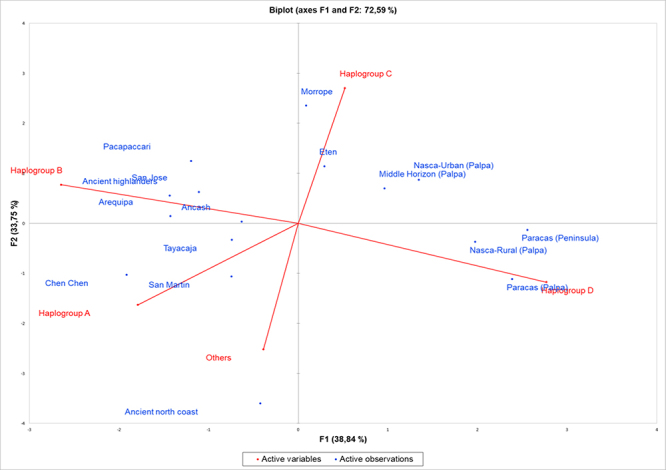
Principal Component Analysis (PCA) for haplogroup frequencies. PCA graph shows the distribution of the populations among the four Amerindian haplogroups in our meta-analysis. Active variables represent the mitochondrial haplogroups, and active observations represent the analyzed populations.

**Figure 5 -:**
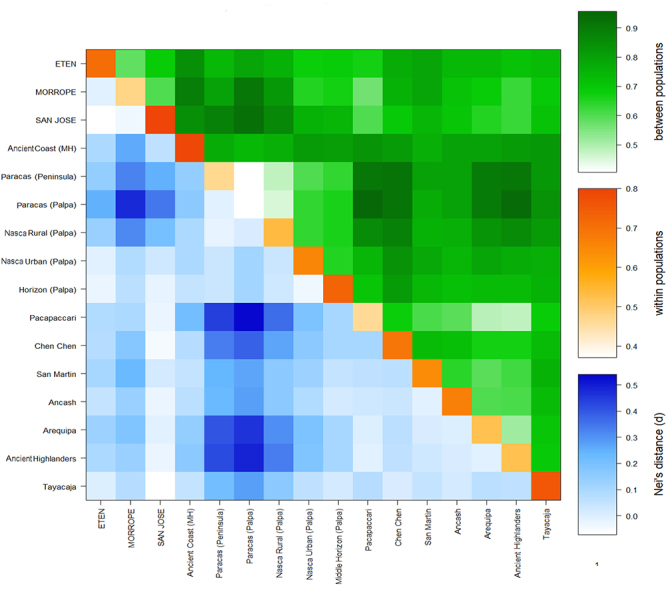
Average number of pairwise differences. Heatmap showing Nei’s distance values against pairwise differences between populations and within populations.

The distribution of haplogroups C and D in the Peruvian coast is remarkable. An estimation of the population structure using principal component analysis (PCA) revealed a shared pattern among coastal populations in contrast to highland populations in which the prevalence of haplogroup B is strong (Figure 4). In addition, AMOVA showed that there was approximately 10% genetic variation among coastal populations, and this value decreased when villages from the north coast were grouped and compared with their counterparts from the south. Low *F*_*st*_ values were obtained (Table 4), which were confirmed by computed Nei’s distance (*d*) (Figure 5).

**Table 4 -:** AMOVA analysis.

Source of variation	Sum of squares	df	Variance components	Percentage of variations	F_ST_	*p*
Coastal populations						
Among groups	4.712	2	0.02789 Va	7.64		
Among population within groups	6.238	6	0.03772 Vb	10.33		
Within populations	51.835	173	0.29962 Vc	82.04	0.17962	< 0.01
Highlands populations						
Among groups	16.104	2	0.06015 Va	15.27		
Among population within groups	11.153	13	0.02336 Vb	5.93		
Within populations	117.360	378	0.31047 Vc	78.81	0.21195	< 0.01

## Discussion

Here, we present new data on the distribution of four Amerindian mitochondrial haplogroups in ancient coastal populations from Peru and sex characterization, using *the AMEL* gene. Our results showed that ancient northern coast civilizations analyzed in this study had a different pattern of mitochondrial haplogroup frequencies when compared with ancient populations from the highlands. This may have been due to admixture of Ecuadorian and Peruvian northern coast individuals, which increases the variability of mitochondrial genetic legacy (Shinoda *et al.* 2006). In addition, new data revealed that the differentiation of northern and southern ancient Andean populations can be explained by cultural and geographical factors leading to population structural differences (Nakatsuka *et al.* 2020). The distribution of haplogroup frequencies in the three populations analyzed in this study demonstrated that the non-exclusive presence of haplogroup B increased after Spanish colonization. Among the four principal Amerindian lineages, haplogroup B has the highest diversity and polymorphism, which can explain the increase in maternal lineages in the highlands of Peru. Moreover, in the Peruvian coast, the maternal heritage is not exclusive for just one mitochondrial haplogroup (Fehren-Schmitz *et al.* 2010). 

An increase in haplogroups C and D frequencies in our samples suggests a possible predominance of these haplogroups in ancient civilizations from Peruvian coastal populations, since these haplogroups had the highest frequency in ancient populations from the Peruvian south coast (Fehren-Schmitz *et al.* 2010).

The data obtained for each population suggested a possible admixture of populations from the coast and the Andes, as we observed the presence of the four Amerindian haplogroups. Indeed, nowadays, mitochondrial diversity in modern populations from the coast is still maintained (Sandoval *et al.* 2018).

In addition, the non-exclusive presence of haplogroup B can be explained by a possible increase in the migration of people from the central Andes to the coast. In fact, the Middle Horizon period (MH: 650-1100 C.E.) was characterized by demographic upheavals that involved the interaction between highland and coastal populations (Valverde *et al.* 2016). 

The samples analyzed in this study belonged to higher periods, from 1200 to 1600 C.E. One example of large changes in the population constitution and social stratification was the colonization of many civilizations settled on the south coast of Peru by the Wari empire (Slovak *et al.* 2009). The impact of Wari imperialism on the genetic structure of several populations was assessed by the analysis of mitochondrial DNA on boundaries from ancient civilizations, which resulted in the identification of the four mitochondrial Amerindian haplogroups and new haplogroups that had not been reported before (Kemp *et al.* 2009; Valverde *et al.* 2016). The presence of these haplogroups supports the hypothesis that an admixture of two populations can lead to novel variations in the human genome over time.

For this reason, genetic analysis of ancient populations is necessary, and our study aims to encourage the study of these civilizations at a molecular level. Unfortunately, sample size in archeological sites does not represent the total number of people who lived in the proximity and could influence haplogroup frequencies, but these results can give us an idea for a possible scenario of maternal legacy that can solve doubts about human diversity and sex characterization on the northern coast of Peru.

## Conclusions

Molecular anthropology studies in the Andes are helping to clarify our understanding of population dynamics in South America. Until now, little was known about the genetic diversity of people from the Lambayeque culture, and we tried to identify a possible maternal line by analyzing boundaries from archeological sites of three localities where this culture settled. We compared our results with previous mitochondrial DNA characterizations of populations from the Peruvian coast to investigate the variation of haplogroups among these populations. Even though our sample is not enough to establish associations between a civilization and an Amerindian haplogroup or among samples, we were able to infer a possible distribution of these haplogroups in the Peruvian coast based on our statistical analysis. For instance, ancient people in Lambayeque presented the four analyzed haplogroups with an increase in haplogroups C and B, which correlated with the findings in other ancient coastal Peruvian populations. Molecular analysis of mitochondrial DNA haplogroups helped to identify polymorphisms shared among samples of ancient human populations that had not been characterized previously. However, this analysis must be complemented with other molecular assays, such as polymorphisms in chromosome Y, to establish an exact kinship among samples.

## Supplementary material

The following online material is available for this article:

Table S1 - Primer sequences for PCR amplification.

Table S2 - Primer sequences for PCR amplification of amelogenin gene.

Table S3 - Sample codes and results for Eten.

Table S4 - Sample codes and results for Morrope.

Table S5 - Sample codes and results for San Jose.

Figure S1 - PCR amplification and restriction enzyme digestion of ancient DNA.
