# Supplementary material for: Molecular characterization of mitochondrial Amerindian haplogroups and the amelogenin gene in human ancient DNA from three archaeological sites in Lambayeque - Peru
Source: Genet Mol Biol. 2020 Nov 25;43(4):e20190265. doi: 10.1590/1678-4685-GMB-2019-0265 (PMC7737099; doi:10.1590/1678-4685-GMB-2019-0265)
Supplement: Table S1 - [file 1415-4757-GMB-43-4-e20190265-s1.pdf]

Genetics and Molecular Biology
https://doi.org/10.1590/1678-4685-GMB-2019-0265

Supplementary Material to "Molecular characterization of mitochondrial Amerindian haplogroups and the amelogenin gene in human ancient DNA from three archaeological sites in Lambayeque – Peru"

Table S1 - Primers sequences for PCR amplification.

Primers	Sequence (5´-3´)	Amplicon size	Haplogroup
L607	CACTGAAAATGTTTAGACGGG	100 bp	A
H707	GGGATGCTTGCATGTGTAAGC		
L8209	CATCGTCCTAGAATTAATTAC	96 bp/85 bp	B
H8304	CTTTACAGTGGGCTCTAGACG		
L13209	CGCCCTTACACAACATGAGATCA	93 bp	C
H13301	GGTTGGTTGAAGCCGCTTGTA		
L5150	CCTACTACTATCTCGCACCTG	67 bp	D
H5217	AGAGGAGGCTGGATGGAACTA		
