# Supplementary material for: Molecular characterization of mitochondrial Amerindian haplogroups and the amelogenin gene in human ancient DNA from three archaeological sites in Lambayeque - Peru
Source: Genet Mol Biol. 2020 Nov 25;43(4):e20190265. doi: 10.1590/1678-4685-GMB-2019-0265 (PMC7737099; doi:10.1590/1678-4685-GMB-2019-0265)
Supplement: Table S2 - [file 1415-4757-GMB-43-4-e20190265-s2.pdf]

Genetics and Molecular Biology
https://doi.org/10.1590/1678-4685-GMB-2019-0265

Supplementary Material to "Molecular characterization of mitochondrial Amerindian haplogroups and the amelogenin gene in human ancient DNA from three archaeological sites in Lambayeque – Peru"

Table S2 - Primers sequences for PCR amplification of Amelogenin gene.

Primer	Sequence (5´-3´)	chrX	chrY
Amel F	GTTTCTTCCCTGGGCTCTGAAAGAATAGTG	120 bp	114 bp
Amel R	TCAGAGCTTAAACTGGGAAGCTG		
