# Supplementary material for: Molecular characterization of mitochondrial Amerindian haplogroups and the amelogenin gene in human ancient DNA from three archaeological sites in Lambayeque - Peru
Source: Genet Mol Biol. 2020 Nov 25;43(4):e20190265. doi: 10.1590/1678-4685-GMB-2019-0265 (PMC7737099; doi:10.1590/1678-4685-GMB-2019-0265)
Supplement: Table S3 - [file 1415-4757-GMB-43-4-e20190265-s3.pdf]

Genetics and Molecular Biology

https://doi.org/10.1590/1678-4685-GMB-2019-0265

Supplementary Material to "Molecular characterization of mitochondrial Amerindian haplogroups and the amelogenin gene in human ancient DNA from three archaeological sites in Lambayeque – Peru"

Table S3 - Sample codes and results for Eten.

Sample	9bp del	Hae III 663	Hinc II 13259	Alu I 5176	Haplogroup	Sex
ENT01	-	-	+	-	D	ud
ENT03	-	-	-	+	C	Female
ENT06	-	-	-	+	C	Male
ENT08	-	+	+	+	A	ud
ENT14	-	-	-	+	C	ud
ENT15	-	-	-	+	C	Female
ENT16	+	-	+	+	B	ud
ENT18	-	-	+	-	D	Male
ENT21	-	-	-	+	C	Male
ENT22	+	-	+	+	B	ud
ENT25	-	-	-	+	C	Male
ENT27	+	-	+	+	B	Female

ud: Undetermined
