# Supplementary material for: Molecular characterization of mitochondrial Amerindian haplogroups and the amelogenin gene in human ancient DNA from three archaeological sites in Lambayeque - Peru
Source: Genet Mol Biol. 2020 Nov 25;43(4):e20190265. doi: 10.1590/1678-4685-GMB-2019-0265 (PMC7737099; doi:10.1590/1678-4685-GMB-2019-0265)
Supplement: Table S4 - [file 1415-4757-GMB-43-4-e20190265-s4.pdf]

Genetics and Molecular Biology
https://doi.org/10.1590/1678-4685-GMB-2019-0265

Supplementary Material to "Molecular characterization of mitochondrial Amerindian haplogroups and the amelogenin gene in human ancient DNA from three archaeological sites in Lambayeque – Peru"

Table S4 - Sample codes and results for Morrope.

Sample	9bp del	Hae III 663	Hinc II 13259	Alu I 5176	Haplogroup	Sex
PM2a	-	-	-	+	C	ud
PM17b	-	-	-	+	C	Female
PM 2b	+	-	+	+	B	ud
PM2c	+	-	+	+	B	Male
PME7	-	-	-	+	C	ud
PM13a	+	-	+	+	B	Male
PM13b	-	-	-	+	C	ud
PM13c	-	-	-	+	C	Female
PM1b	+	-	+	+	B	ud
PM14	-	-	-	+	C	Female
PM4	-	-	-	+	C	Female
PM1	-	-	-	+	C	Male
PM7	-	-	-	+	C	ud
PMU5	+	-	+	+	B	ud
PME3	-	-	-	+	C	ud

ud: Undetermined
